# How to Avoid the Taste of Medicine

**Published:** 1887-01-01

**Authors:** A. F. Durham

**Affiliations:** Georgia


### HOW TO AVOID THE TASTE OF MEDICINE.

BY A. F. DURHAM, M. D., OF GEORGIA.

*Editor of Record:*

Dear Sir:—While sitting here ruminating over things generally, an article or two that appeared in your journal sometime back on "How to Take Medicine Without Tasting It," occurred to me, and to pass away a few leisure moments pleasantly I thought I would write a discovery of mine on the same. It is my discovery so far as I know, never having seen or read of the plan. The idea first occurred to me after reading the articles above referred to, while studying the mixed functions of smell and taste, that is, their relation to each other. When a person has a very bad cold he can't taste well, and on tasting any substance, after touching it to the tip of the tongue, the lips are always brought together, that is, the taster always "smacks" his lips. Well, that reflection led me to make the following very simple experiment, and I must say I was not much disappointed in finding it almost, if not entirely, a perfect success. It is simply to double the tongue up like a water-lapping animal, and while the tongue is troughed, or partially folded back on itself, drop whatever substance you experiment with on the middle of the tongue—quinine or morphia, for instance—and without letting the tongue move or coming in contact with the teeth, lips or gums, wash it down with a little water, and if you have never tried it, you will be perfectly astonished how little taste will be perceived. You may drop anything on the tongue dry, and allow it to remain almost any length of time, without detecting the slightest taste.

You will find that it requires a good deal of care at first to make the experiment, as there is at first some difficulty in swallowing without moving the tongue, but you will soon catch the art. Try it, and if you find the above to be true, you can publish it if you think it worthy. This plan can not be applied to large doses of liquids, as oil, etc.

